# Populations at risk: conservation genetics of kangaroo mice (*Microdipodops*) of the Great Basin Desert

**DOI:** 10.1002/ece3.637

**Published:** 2013-06-26

**Authors:** John J Andersen, David S Portnoy, John C Hafner, Jessica E Light

**Affiliations:** 1 Department of Wildlife and Fisheries Sciences, Texas A&M University210 Nagle Hall, College Station, Texas, 77843-2258; 2 Department of Biological Sciences, Louisiana State University202 Life Sciences Building, Baton Rouge, Louisiana, 70808; 3 Moore Laboratory of Zoology and Department of Biology, Occidental CollegeLos Angeles, California, 90041; 105 Franklin Avenue, Fortuna, California, 95540

**Keywords:** Effective population size, Great Basin Desert, historical biogeography, kangaroo mice, *Microdipodops*, microsatellites, *N*_e_, population genetics

## Abstract

The Great Basin Desert of western North America has experienced frequent habitat alterations due to a complex biogeographic history and recent anthropogenic impacts, with the more recent alterations likely resulting in the decline of native fauna and flora. Dark (*Microdipodops megacephalus*) and pallid (*M. pallidus*) kangaroo mice are ecological specialists found within the Great Basin Desert and are potentially ideal organisms for assessing ecosystem health and inferring the biogeographic history of this vulnerable region. Herein, newly acquired nuclear-encoded microsatellite loci were utilized to assess patterns of variation within and among spatially discrete groups of kangaroo mice and to evaluate gene flow, demographic trends, and genetic integrity. Results confirm that there are at least three genetically distinct units within *M. megacephalus* and two such units within *M. pallidus*. The three units of *M. megacephalus* appear to have different demographic histories, with effectively no gene flow among them since their divergence. Similarly, the two units of *M. pallidus* also appear to have experienced different demographic histories, with effectively no gene exchange. Contemporary effective population sizes of all groups within *Microdipodops* appear to be low (<500), suggesting that each genetic lineage may have difficulty coping with changing environmental pressures and hence may be at risk of extirpation. Results of this study indicate that each *Microdipodops* group should be recognized, and therefore managed, as a separate unit in an effort to conserve these highly specialized taxa that contribute to the diversity of the Great Basin Desert ecosystem.

The Great Basin Desert of western North America has experienced frequent habitat alterations due to a complex biogeographic history and recent anthropogenic impacts, with the more recent alterations likely resulting in the decline of native fauna and flora. Herein, newly acquired nuclear-encoded microsatellite loci were utilized to assess patterns of variation within and among spatially discrete groups of the dark (*Microdipodops megacephalus*) and pallid (*M. pallidus*) kangaroo mouse, and to evaluate gene flow, demographic trends, and genetic integrity. Results of this study indicate that each *Microdipodops* group should be recognized, and therefore managed, as a separate unit in an effort to conserve these highly specialized taxa that contribute to the diversity of the Great Basin Desert ecosystem (photo credit J. C. Hafner).

## Introduction

The Great Basin Desert of western North America is characterized by a series of alternating islands of mountain ranges and desert basins (Fiero 17) that formed a backdrop to a dynamic biogeographic history (Davis 11). The glacial–interglacial cycles of the Pleistocene (Riddle 57) and the associated rise and fall of pluvial lakes (Benson 7), shifting climatic patterns (Atvens 2), and floristic transitions (Reveal 55) have caused numerous habitat alterations throughout the Great Basin Desert. More recently, anthropogenic habitat alterations (e.g., introduction of nonnative plant species, increased wildfires, and cultivation and irrigation) have also plagued the area (Hafner and Hafner 24). These alterations have caused a significant loss of available habitat and subsequent reduction in the abundance of native fauna and flora. For example, representatives of the rodent genus *Microdipodops* (kangaroo mice; family Heteromyidae) have become increasingly rare members of the Great Basin Desert community (Hafner and Upham 25).

Two species of *Microdipodops* are currently recognized: the dark kangaroo mouse (*M. megacephalus*) and the pallid kangaroo mouse (*M. pallidus*). Both species are sand-obligate endemics to the Great Basin Desert and, as such, are highly specialized to survive in an extreme environment (Hafner 23). In fact, morphology within the genus is extremely conserved with only slight differences between sibling taxa (Hafner et al. 28). Given their ecological specializations, these small nocturnal rodents likely serve as indicator species of healthy, sandy desert habitats of the Great Basin. Field observations, however, have concluded that the numbers of both *M. megacephalus* and *M. pallidus* are dwindling (Hafner 23; Hafner and Hafner 24; Hafner et al. 28; Hafner and Upham 25), as is the case for other flora and fauna distributed across the Great Basin Desert (Brussard et al. 9). However, both *Microdipodops* species are listed as “Least Concern” by the International Union for Conservation of Nature (IUCN) and are not protected (Linzey and Hammerson 43; Linzey et al. 44). Given their decreasing numbers, this listing is outmoded and management of kangaroo mice, along with other Great Basin Desert organisms, will be necessary to help preserve this threatened ecosystem.

**Figure 1:**
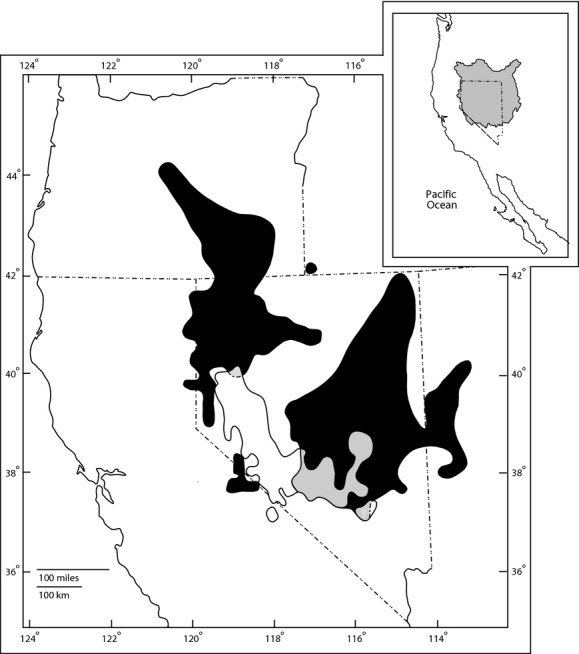
Geographic distribution of kangaroo mice in the Great Basin Desert of the western United States. Dark kangaroo mice (*Microdipodops megacephalus*) are in black, pallid kangaroo mice (*Microdipodops pallidus*) are in white (outlined in black), and areas where their ranges overlap are in gray. The Great Basin Desert is depicted as the shaded area in the inset map of western North America and includes the outline of the state of Nevada for orientation.

**Figure 2:**
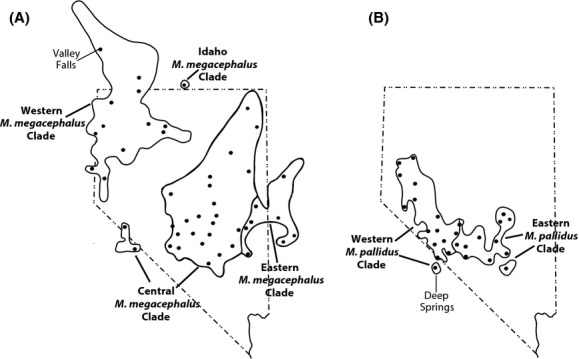
Detailed geographic distributions of mtDNA clades within dark and pallid kangaroo mice. (A) Geographic distribution of *Microdipodops megacephalus*, with labels corresponding to mtDNA clades (eastern, central, western, and Idaho) discussed in prior studies (Hafner et al. 27; Hafner and Upham 25). The genetically distinct Valley Falls subunit (which is nested within the western clade; Hafner and Upham 25; Light et al. 42) also is labeled. (B) Geographic distribution of *M. pallidus*, with labels corresponding to mtDNA clades (eastern and western) from prior studies (Hafner et al. 28); the isolated Deep Springs locality (which is nested within the western clade; Hafner et al. 28) also is labeled. Dots indicate exact collecting localities for specimens used in this study and identified in Hafner et al. (28) and Hafner and Upham (25); outline of the State of Nevada provides proper orientation.

*Microdipodops megacephalus* and *M. pallidus* have unique habitat associations within the Great Basin Desert. Although their distributions overlap (Fig. 1), these species show differential niche specializations. *Microdipodops megacephalus* is primarily restricted to sandy soils with gravel overlay and found in association with sagebrush and/or rabbit brush (Hafner and Upham 25; and references therein); whereas *M. pallidus* prefers greasewood and fine soils with no gravel overlay (Hafner 23; and references therein). Ancient and current habitat alterations have led to fragmented distributions for both species such that current intraspecific ranges are disjunct (Figs. 1, 2), separated either by geological barriers (e.g., mountain ranges) or unsuitable habitat (Hafner et al. 28; Hafner and Upham 25).

These unique, fragmented distributions and ecological specializations have made kangaroo mice the recent subjects of several studies that used mitochondrial DNA (mtDNA) gene regions to elucidate the biogeographic history of the Great Basin Desert (Hafner et al. 27, 28; Hafner and Upham 25; Light et al. 42). These studies identified and supported four distinct mtDNA clades in *M. megacephalus* (the eastern, central, western, and Idaho clades; Fig. 2A) and two distinct mtDNA clades in *M. pallidus* (the eastern and western clades; Fig. 2B). While the identification of genetically discrete units within each species is important, additional analyses using fast-evolving nuclear markers, such as microsatellites, are necessary to verify the results of the mtDNA data. These markers also can help to estimate parameters for conservation and management of these specialized taxa; for example, estimates such as rates of gene flow and effective population sizes for *Microdipodops* are currently unknown. Lastly, examination of multiple markers can facilitate a better understanding of genetic lineages within a species (Avise 3), especially as these markers may have different evolutionary histories (e.g., Yang and Kenagy 69).

Herein, we use microsatellite markers to provide an assessment of nuclear variation within each *Microdipodops* species and to test the findings from previous studies which used mtDNA sequence data (Hafner et al. 27, 28; Hafner and Upham 25; Light et al. 42). We hypothesize that microsatellite markers will support discrete genetic units within each *Microdipodops* species and uncover the same geographic groups found in previous studies. Due to the wealth of information available regarding *Microdipodops* biogeography, population-level analyses are performed on microsatellite data with samples disaggregated into geographic regions identified in previous studies (Fig. 2) and results are interpreted in reference to Great Basin biogeography. These findings will help to identify evolutionarily significant units and address issues of management, conservation, and desert biogeography that can be applied to other flora and fauna of the threatened Great Basin Desert.

## Materials and Methods

### Specimens examined

A total of 184 specimens of *M. megacephalus* from 46 localities, and a total of 105 specimens of *M. pallidus* from 27 localities, were used in this study (Table A1; terminology follows Hafner et al. 28; Hafner and Upham 25; and Light et al. 42). The majority of these specimens were collected for use in prior studies: the *M. megacephalus* specimens were collected between 1975 and 1976, 1999 and 2007, and in 2011. The *M. pallidus* specimens were collected between 1999 and 2005, with one individual sampled in 1975. Any newly collected specimens were collected according to procedures approved by the Occidental College's Animal Care and Use Committee and the American Society of Mammalogists (Sikes et al. 60). All tissues were stored in a −80°C freezer.

For many of the analyses, populations were defined by grouping specimens together within each species based on geography. These geographic units correspond to previously identified mtDNA clades (Fig. 2) and subclades. Previous studies recognized four geographic units within *M. megacephalus* (Hafner and Upham 25; Light et al. 42): the eastern geographic unit (*n *=* *49) with two subunits (eastern subunit, *n *=* *25; western subunit, *n *=* *24), the central geographic unit (*n *=* *69) with two subunits (central subunit, *n *=* *19; western subunit, *n *=* *50), the western geographic unit (*n *=* *62) with one subunit (Valley Falls, *n *=* *9), and the Idaho geographic unit (*n *=* *4). Two geographic units were recognized within *M. pallidus* (Hafner et al. 28; Light et al. 42): the eastern geographic unit (*n *=* *42) with two subunits (eastern subunit, *n *=* *18; south-central subunit, *n *=* *24), and the western geographic unit (*n *=* *63) with one subunit (Deep Springs, *n *=* *10).

### Laboratory methods

DNA extracts were available from previous studies (Hafner et al. 27, 28; Hafner and Upham 25). When original extractions were depleted, DNA was extracted from liver or kidney tissues as described by Hafner et al. (27). Seventeen polymorphic microsatellite loci, developed for *Microdipodops* by Lance et al. (40), were tested on preliminary samples and loci that did not reliably amplify were subsequently removed. Polymerase chain reactions (PCR) followed Boutin-Ganache et al. (8) and contained three primers: a forward primer with an attached 16-bp tail sequence (5'-CAGTCGGGCGTCATCA-3'), a 6-Fam or Hex (Dye Set D, Applied Biosystems, Foster City, CA) labeled tail sequence (defined above), and an unlabeled reverse primer. Amplified DNA from each PCR reaction was combined with a 400 HD Rox size-standard DNA ladder (Applied Biosystems) and electrophoresed on 6% polyacrylamide gels using an ABI PRISM 377 DNA Sequencer (Applied Biosystems). Sizes of microsatellite fragments were visualized in GENESCAN v. 3.1.2 (Applied Biosystems) and assessed using GENOTYPER v. 2.5 (Applied Biosystems).

### Data analysis

Each microsatellite locus was tested for conformance to the expectations of Hardy–Weinberg equilibrium (HWE) using Genepop v. 4.0 (Raymond and Rousset 54; Rousset 58). Significance was assessed at the 0.05 level, using exact tests with 20 batches and 5000 iterations per batch, and sequential Bonferroni adjustment was used to correct for multiple testing (Rice 56). Loci that differed significantly from the expectations of HWE were assessed either by rescoring gels and/or rerunning PCR to determine if genotyping error caused spurious results. Genepop also was used to calculate the expected and observed numbers of heterozygotes, test for genotypic disequilibrium, and calculate gene frequencies when null alleles were present. Number of alleles and allelic richness (i.e., number of alleles per locus, averaged over the smallest population) for each locus were calculated with Fstat v. 2.9.3.2 (Goudet 21).

Population structure within each species was first assessed to test for genetic homogeneity with an analysis of molecular variance (AMOVA; Excoffier et al. 15) implemented in Arlequin v. 3.5.1.2 (Excoffier and Lischer 14). AMOVA was performed in a hierarchical fashion with populations grouped a priori by geographic unit, and significance was assessed by 10,000 randomization replicates. Using the same assortment of geographic units, *F*_ST_ and *R*_ST_ (a *F*_ST_ analog assuming a stepwise mutation model; Slatkin 61) statistics were estimated with Arlequin, and significance at the 0.05 level was assessed by permuting individuals between samples 10,000 times. Allele size permutation tests were performed to compare *F*_ST_ and *R*_ST_ statistics using SPAGeDi 1.4 (Hardy and Vekemans 31).

The Bayesian multilocus clustering algorithm found in Structure v. 2.3.3 (Pritchard et al. 52) was used to examine fine-scale population structure without defining populations a priori. Analyses were run in a hierarchical manner, first within *M. megacephalus* and *M. pallidus*, and then within each geographic unit; the eastern, central, western, and Idaho geographic units of *M*. *megacephalus*, and the eastern and western geographic units of *M. pallidus*). The population admixture model was used with 10 replicate runs from *K *=* *1 to *K *=* *10, where *K* is a user-defined number of clusters. Each run consisted of a burn-in of 10,000 steps followed by 100,000 additional steps. To evaluate the most likely *K* value, Structure Harvester (Earl and vonHoldt 12) was used to graph both the mean estimated ln Prob (Data) and Δ*K* (change in ln Prob (Data) between successive *K* values) as suggested by Evanno et al. (13).

Migrate-N v. 3.2.1.6 (Beerli and Felsenstein 6; Beerli 5) was used to estimate theta (*θ*;* θ *= 4*N*_eLT_*μ*, where *N*_eLT_ is the long-term effective population size and *μ* is the per-generation mutation rate) and *M* (mutation-scaled migration rate) among geographic units within each species using Bayesian inference. Due to the small sample size of the *M. megacephalus* Idaho geographic unit (*n *=* *4), it was excluded from the analysis. Theta was estimated to detect if there were significant differences in *N*_eLT_ among geographic units, while *M* was used to quantify average, long-term gene flow between geographic units. Preliminary runs were performed to estimate priors for *M* and *θ* for final runs. Final runs were executed in replicate at different starting points and parameter estimates were examined to ensure chain mixing and convergence. For *M. megacephalus*, runs consisted of three long chains, geometric heating, and a burn-in of 100,000 steps followed by 1,000,000 steps with a tree recorded every 100 steps, resulting in 10,000 trees sampled. For *M. pallidus,* runs consisted of one long chain and a burn-in of 10,000 steps followed by 100,000 steps with a tree recorded every 100 steps, resulting in 1000 trees. In all analyses, effective sample sizes were >50.

IMa (Hey and Nielsen 33) also was used to determine *θ* and *M* among geographic units of *M. megacephalus* and *M. pallidus* in a pairwise manner. IMa differs from Migrate-N in that it takes coancestry into account when looking at migration and it assumes there is one ancestral panmictic population for each extant population. This assumption allows the estimation of the ancestral effective population size and time since divergence (*t*), where a positive *t* value would indicate divergence and a value that peaked at or very close to zero would reveal no divergence (Portnoy et al. 51). Additionally, while both Migrate-N and IMa use the Metropolis-Hastings criterion, IMa incorporates a Metropolis-Coupled version of the algorithm which enables multiple heated chains to search the parameter space simultaneously and can provide a more thorough mixing of chains (Hey and Rasmus 34). Preliminary runs were performed to assess whether the heating conditions and *M*,* θ*, and *t* priors were appropriate for the data set. Final runs consisted of 50 chains with geometric heating (to ensure acceptable chain mixing and low autocorrelations) and a burn-in of at least 1,000,000 generations followed by at least 90,000 generations (resulting in effective sample sizes >50). Final runs were executed in replicate with different starting seeds to ensure convergence, and the R package BOA (Smith 62) was used to visually assess convergence of posterior distributions and examine autocorrelation at different lags to determine appropriate run time.

Bayesian inference of immigration rates (BIMr; Faubet and Gaggiotti 16) was used to estimate current rates of gene exchange among geographic units, thus facilitating comparison with long-term estimates of *M* from Migrate-N and IMa. Preliminary pilot runs (each at a length of 2000 steps) were executed to provide a rough estimation of starting points for final runs. Replicate runs consisted of a burn-in of 20,000 iterations, followed by an additional 100,000 and 60,000 iterations for *M. megacephalus* and *M. pallidus*, respectively. The R package BOA (Smith 62) was used to examine autocorrelation at different lags to determine appropriate run time and visually assess convergence of posterior distributions. Density functions were analyzed and the mode (point estimate) and 95% highest posterior density interval (HPDI) were noted.

The program LdNe (Waples and Do 67) was used to estimate contemporary effective population size (*N*_e_) via the modified linkage disequilibrium approach (Hill 35; Waples 66) for each geographic unit within *M. megacephalus* and *M. pallidus*. Effective population size is a crucial parameter in conservation and wildlife management because of its influence on population viability and ability to predict extinction risk (Luikart et al. 45). LdNe assumes that the correlation of unlinked alleles at unlinked loci arises from genetic drift in an isolated population (Hill 35; Wang 202) with estimates reflecting the number of parents that contributed to the sample (Waples 65). *Microdipodops* are semelparous with a generation time of 1 year (Hall 30), meaning that estimates are of contemporary *N*_e_ rather than effective number of breeders (*N*_b_; Jorde and Ryman 39; Waples 65). Because allele frequencies close to 0 or 1 can skew *N*_e_ results (Waples 66; Portnoy et al. 50), alleles that had a frequency of <2% were omitted from analyses. For all analyses, a random mating model was assumed and 95% jackknife confidence intervals were assessed (Waples 66).

Extended Bayesian Skyline Plots (EBSP; Heled and Drummond 32) were used to estimate *N*_e_ through time within each geographic unit of *M. megacephalus* and *M. pallidus*. EBSP differs from other demographic analyses in that it estimates the population function directly from the data. Furthermore, unlike estimates of contemporary *N*_e_, EBSP estimates *N*_e_ through a coalescent approach, and *N*_e_ estimates can therefore be used to find varying historic demographic changes across lineages. Each geographic unit was analyzed individually (although individuals from the *M. megacephalus* Idaho geographic unit were excluded due to small sample size). MSVAR v. 1.3 (Beaumont 4) was used to estimate the average mutation rate for all loci within each geographic unit. Uniform rate analyses were run using a strict molecular clock following a stepwise mutation model. A minimum of two runs of 1 billion generations were performed, with a tree recorded every 40,000 steps after a 10% burn-in. Effective sample sizes and number of population size changes were assessed in Tracer v 1.5 (Rambaut and Drummond 53). Population size data were plotted using R (R Development Core Team 201).

## Results

Of the 17 initial polymorphic loci screened, 11 and 10 loci amplified successfully and were used in the population genetic analyses of *M. megacephalus* and *M. pallidus*, respectively (summary data available in Tables A2, A3). One locus in *M. megacephalus* was monomorphic in the western and Idaho geographic units, but polymorphic in the eastern and central geographic units. After correction for multiple tests, genotypes at two loci (*Mime11* and *Mime32*) in *M. pallidus* from the western geographic unit deviated significantly from the expectations of HWE. This was due to the isolated population from Deep Springs (Fig. 2B) where homozygote excess occurred at both loci. When Deep Springs was excluded from analysis, all loci conformed to the expectations of HWE. Less computationally intensive analyses (e.g., AMOVA, Structure, pairwise *R*_ST_) were run including and excluding the two deviated loci, and there was no difference in the results. Therefore, results reported in this study included all loci that amplified successfully.

Allele size permutation tests indicated that *R*_ST_ values were consistently significantly greater than *F*_ST_ values, indicating that *F*_ST_ may be underestimating actual values of genetic structure (Table A4; Hoffman et al. 36). Thus, only *R*_ST_ results are presented here. AMOVA revealed significant population structure among geographic units and among subunits within geographic units in both species (*P *<* *0.001; Table 1). Pairwise estimates of *R*_ST_ among geographic units within *M. megacephalus* ranged from 0.16 (*P *<* *0.0001; eastern and central geographic units) to 0.61 (*P *<* *0.0001; eastern and Idaho geographic units), and the *R*_ST_ estimate between *M. pallidus* geographic units was 0.89 (*P *<* *0.0001). AMOVA analyses focusing on specific geographic units showed a significant component of variation among subunits in the *M. megacephalus* western geographic unit (Valley Falls and the rest of the western unit) and the *M. pallidus* western geographic unit (Deep Springs and the rest of the western unit; pairwise *Φ*_ST_ = 0.17 and 0.11, respectively, *P *<* *0.0001). The remaining subunit analyses resulted in nonsignificant variation, possibly due to small sample sizes or lack of physical isolation.

**Table 1:** AMOVA among the four geographic units of *Microdipodops megacephalus* and the two geographic units of *M. pallidus*

Source of variation	Variance components	% of variance	Φ	*P*
*M. megacephalus*				
Among geographic units	48.6338	51.87	0.5187	*P *<* *0.0001
Among populations within geographic units	8.9543	9.55	0.1984	*P *<* *0.0001
Within individuals	36.1794	38.58	0.61416	*P *<* *0.0001
*M. pallidus*				
Among geographic units	263.45782	88.79	0.8879	*P *<* *0.001
Among populations within geographic units	8.24233	2.78	0.2478	*P *<* *0.0001
Within individuals	25.01905	8.43	0.9157	*P *<* *0.0001

**Figure 3:**
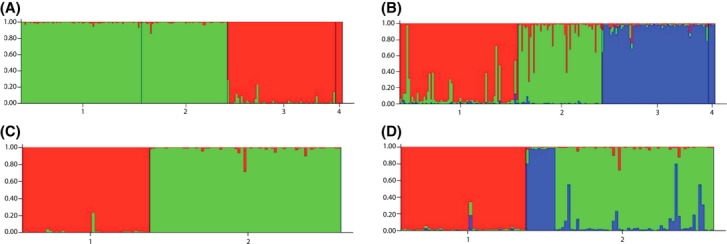
Structure bar plots. (A) *Microdipodops megacephalus* for *K *=* *2 (1 = central geographic unit, 2 = eastern geographic unit, 3 = western geographic unit, 4 = Idaho geographic unit). (B) *Microdipodops megacephalus* for *K *=* *3 (1 = central geographic unit, 2 = eastern geographic unit, 3 = western geographic unit, 4 = Idaho geographic unit). (C) *Microdipodops pallidus* for *K = *2 (1 = eastern geographic unit, 2 = western geographic unit). (D) *Microdipodops pallidus* for *K *=* *3 (1 = eastern geographic unit, 2 = western geographic unit; the area in blue corresponds to Deep Springs; Fig. 2).

Structure analyses revealed that *K *=* *3 was the most likely number of clusters for both *M. megacephalus* and *M. pallidus* (when plotting ln Prob (Data); Fig. 3). The 3 clusters of *M. megacephalus* corresponded to the (1) eastern, (2) central, and (3) western/Idaho geographic units. The 3 clusters of *M. pallidus* corresponded to the (1) eastern geographic unit, (2) Deep Springs subunit, and (3) the remainder of the western geographic unit for *M. pallidus*. Additional bar plots with increasing number of clusters also were analyzed in case discrete populations could be distinguished. However, population structure became less resolved with *K *>* *3. Results from the Δ*K* metric suggested by Evanno et al. (13) indicated that *K *=* *2 was the most strongly supported (Δ ln Prob (Data) = 388.02 and 551.32 for *M. megacephalus* and *M. pallidus*, respectively), while *K *=* *3 was the next most strongly supported (Δ ln Prob (Data) = 106.02 and 24.86 for *M. megacephalus* and *M. pallidus*, respectively). The 2 clusters were an eastern/central and western/Idaho group for *M. megacephalus* and an eastern and western group for *M. pallidus*. When individual geographic units were analyzed separately, *K *=* *1 was the most likely number of clusters of nuclear variation for all units with the exception of the *M. pallidus* western geographic unit, where *K *=* *2 was most likely number of clusters (corresponding to Deep Springs and the rest of the western geographic unit).

Gene flow estimates from Migrate-N for *M. megacephalus* were low, with modal values for *M* ranging from 0 (eastern ↔ western and central → western) to 0.05 (eastern → central; Table 2). While estimates of *M* from central → eastern were higher (0.18), the 2.5% and 97.5% bounds were 0.01 and 0.92, respectively. With such a large confidence interval it was therefore unclear how much long-term gene exchange was occurring between these two geographic units. Estimates of *θ* were not statistically different among geographic units (Table 3).

**Table 2:** Values of *M* (mutation-scaled migration rate) for *Microdipodops* geographic units generated in Migrate-N and IMa

	Eastern unit	Central unit	Western unit
*M. megacephalus*			
*M* (Migrate-N)			
Eastern unit	–	0.05 (0.0–0.36)	0.0 (0.0–0.04)
Central unit	0.18 (0.01–0.92)	–	0.0 (0.0–0.08)
Western unit	0.0 (0.0–0.01)	0.02 (0.0–0.11)	–
*M* (IMa)			
Eastern unit	–	0.07 (0.01–0.32)	0.01 (0.01–0.13)
Central unit	0.01 (0.01–0.14)	–	0.01 (0.01–0.07)
Western unit	0.01 (0.01–0.10)	0.01 (0.01–0.11)	–
	Eastern unit	Western unit	
*M. pallidus*			
*M* (Migrate-N)			
Eastern unit	–	0.01 (0.0–0.05)	
Western unit	0.03 (0.0–0.08)	–	
*M* (IMa)			
Eastern unit	–	0.09 (0.03–0.23)	
Western unit	0.04 (0.01–0.16)		

Values in parentheses are 95% confidence intervals. Directionality of gene flow is read from geographic units on the left being the source populations while geographic units on top are the recipient populations.

**Table 3:** Values of Theta (*θ*; generated in Migrate-N and IMa) and contemporary effective population size (*N*_e_; mode and putative 95% jackknife confidence intervals; generated in LdNe) for *Microdipodops* geographic units

	2.5%	Mode	97.5%	Parental *N*_e_
*M. megacephalus* (Migrate-N; IMa)				
Eastern unit	9.28; 12.76	12.18; 24.5	16.23; 42.19	378.1 (166.3–∞)
Central unit	13.03; 11.77	17.08; 22.46	21.88; 39.58	341.0 (179.8–1914.5)
Western unit	9.93; 6.61	13.98; 14.16	19.36; 25.49	213.2 (108.8–1385.9)
*M. pallidus* (Migrate-N; IMa)				
Eastern unit	8.26; 5.15	11.35; 8.09	15.84; 11.04	287.9 (95.1–∞)
Western unit	11.52; 6.63	15.58; 11.05	20.88; 15.46	128.3 (80.5–270.3)

Estimates of *M* from Migrate-N within *M. pallidus* were 0.03 (eastern → western) and 0.01 (western → eastern; Table 2). The estimates were not significantly different from zero and the upper bounds were 0.08 and 0.05, respectively. Estimates of *θ* for the *M. pallidus* eastern and western geographic units were not statistically different (Table 3).

Results from IMa analysis of *M. megacephalus* revealed that the lower bound of time since divergence (*t*) did not include zero for the eastern, central, and western geographic units, indicating divergence from an ancestral, panmictic population. Estimates for *t* were quite low (ranging from ca. 8600–13,900 years before present) with large confidence intervals (often over hundreds of thousands of years; data available upon request). Estimated long-term gene flow (*M*) from IMa was very small, with a lower confidence interval and mode for all three groups of 0.01 (with one exception of a modal estimate for *M* of 0.07 from eastern → central; Table 2). Estimates of *M* from eastern → central had much tighter confidence intervals compared to Migrate-N results, suggesting greater precision in the IMa analysis. Theta estimates for the eastern, central, and western *M. megacephalus* geographic units were not statistically different (Table 3).

IMa analysis of *M. pallidus* revealed that the lower bound of *t* did not include zero indicating that the eastern and western geographic units had diverged from an ancestral, panmictic population. The estimate for *t* was low (ca. 9500 years before present), with a rather large confidence interval (spanning nearly 500,000 years). Estimated rates of gene flow for eastern → western and western → eastern were 0.09 and 0.04, respectively (Table 2). These estimates of *M* indicate no or extremely low levels of possible long-term gene exchange. Theta estimates for the eastern and western *M. pallidus* geographic units were not statistically different (Table 3).

Estimates of current gene flow rates from BIMr for geographic units of *M. megacephalus* showed modal values from 2.19 × 10^−11^ to 3.56 × 10^−16^ (Table 4). Such small estimates suggest effectively no gene exchange across geographic units within the last generation. Modal estimates for the geographic units of *M. pallidus* (Table 4), while larger than those for *M. megacephalus*, similarly suggest effectively no current gene flow between the eastern and western geographic units within the last generation.

**Table 4:** Modal values and their 95% quartiles for rates of current gene flow from the previous generation (from BIMr analyses) for *Microdipodops* geographic units

Sample	2.5%	Mode	97.5%
*M. megacephalus*			
Eastern → Central unit	1.59 × 10^−5^	2.86 × 10^−16^	7.8 × 10^−3^
Eastern → Western unit	2.74 × 10^−16^	1.82 × 10^−15^	4.8 × 10^−4^
Central → Eastern unit	4.71 × 10^−9^	3.56 × 10^−16^	1.15 × 10^−6^
Central → Western unit	5.63 × 10^−9^	1.82 × 10^−15^	9.18 × 10^−7^
Western → Eastern unit	2.43 × 10^−9^	2.19 × 10^−11^	5.93 × 10^−10^
Western → Central unit	1.26 × 10^−12^	2.19 × 10^−11^	5.9 × 10^−10^
*M. pallidus*			
Eastern → Western unit	3.4 × 10^−3^	2.33 × 10^−3^	4.54 × 10^−2^
Western → Eastern unit	2.3 × 10^−4^	1.7 × 10^−3^	3.24 × 10^−2^

Point estimates of contemporary *N*_e_, as well as minimum and maximum estimates (based on 95% confidence intervals obtained by jackknifing), for *M. megacephalus* and *M. pallidus* are presented in Table 3. For all populations of both species, point estimates were <500. Minimum estimates of *N*_e_ (based on 95% confidence intervals), which may serve as a conservative estimate for wildlife management (Waples and Do 68), ranged from 108.8 individuals (western geographic unit) to 179.8 (central geographic unit) in *M. megacephalus*, and were 95.1 and 80.5 in the eastern and western geographic units of *M. pallidus*, respectively. The eastern unit of *M. megacephalus* and the eastern unit of *M. pallidus* were the only geographic units with upper limits of infinity (∞).

**Figure 4:**
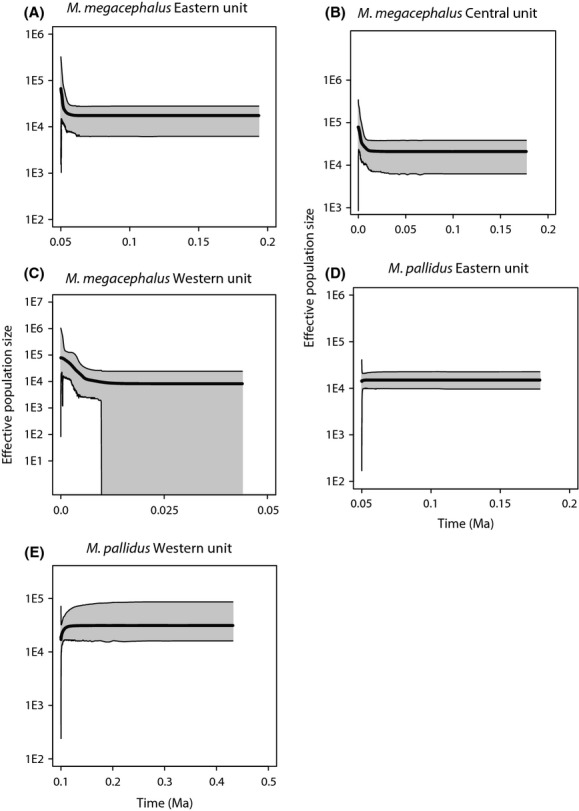
Extended Bayesian Skyline Plots (EBSP) for each geographic unit within dark and pallid kangaroo mice based on microsatellite data. The gray shading corresponds to the 95% highest posterior density (HPD) around the mean *N*_e_ (thick black line). For all plots, *x*-axis values are millions of years before present (Ma) and *y*-axis values are estimates of effective population size (*N*_e_).

Mutation rates estimated by MSVAR averaged 2.40 × 10^−4^, 2.75 × 10^−4^, 2.45 × 10^−4^, 3.23 × 10^−4^, and 3.89 × 10^−4^ for the *M. megacephalus* eastern, central, and western geographic units, and the *M. pallidus* eastern and western geographic units, respectively. EBSP results showed that the three *M. megacephalus* geographic units might have undergone a recent population expansion, whereas the *M. pallidus* eastern unit remained fairly constant and the western unit underwent a recent population contraction (Fig. 4). None of these results, however, were significant (Fig. 4). While these results seem to contradict one another, it is important to note that EBSP is generating long-term estimates of *N*_e_ while LDN_e_ is generating estimates of contemporary N_e_. The two estimates may therefore differ because (a) the time periods to which the two effective size estimates apply are not necessarily concordant (Waples 65) and (b) long-term estimates are more affected by long-term gene flow, even from extinct demes, than contemporary estimates and may reflect global effective size rather than local (Schwartz et al. 59). One additional parameter that can be estimated from EBSP is the number of population size changes since time of coalescence. When examining the 95% HPD of demographic population size changes, we failed to reject a constant population size in the *M. megacephalus* eastern and central geographic units and both *M. pallidus* geographic units (population size changes ranged from 0 to 3 in all units, except the *M. pallidus* eastern unit which ranged from 0 to 2). We could, however, reject a constant population size in the *M. megacephalus* western geographic unit (population size changes ranged from 1 to 3).

## Discussion

Microsatellite markers reveal that *M. megacephalus* and *M. pallidus* are comprised of multiple genetically distinct units within the Great Basin Desert. Primary population genetic analyses (AMOVA and pairwise *R*_ST_) support genetic heterogeneity within each species and Structure analyses revealed that *K *=* *3 was the most likely number of clusters for both *M. megacephalus* and *M. pallidus* (Fig. 3). Although the Δ*K* metric indicated that *K *=* *2 was the most likely number of clusters for *M. megacephalus* and *M. pallidus*, this method is more conservative and often underestimates *K* with insufficient sample sizes (Evanno et al. 13). Thus, the Structure results are more appropriate for the *Microdipodops* data examined in this study. The genetic clusters identified here correspond to the mtDNA clades identified in previous studies (Hafner et al. 27, 28; Hafner and Upham 25; Light et al. 42). The only exception is the lack of recognition of a cluster corresponding to the Idaho clade within *M. megacephalus*. Hierarchical Structure analyses failed to reveal two genetic clusters within the *M. megacephalus* western/Idaho cluster; however, increasing *K* to 3 showed a clearly diverged Idaho cluster with an unresolved western cluster (results available upon request). Increasing the number of individuals from the Idaho clade in future studies would probably tease it apart from the western cluster (Evanno et al. 13; Hale et al. 29). In addition to supporting distinct genetic lineages within each *Microdipodops* species, this study provides an in-depth assessment of parameters important for conservation and management, including patterns of current and historical connectivity (gene flow), effective population sizes, and demographic history. Results of this study therefore provide information that can be used for management strategies and conservation efforts specific to each evolutionarily significant unit within the Great Basin Desert.

Significant population structure detected within *M. megacephalus* supports the perspective that kangaroo mice found in the eastern, central, and western units are, at minimum, distinct populations (specimens representing the Idaho clade of *M. megacephalus* could not be analyzed rigorously due to a small sample size). In agreement with previous phylogenetic analyses, population genetic analyses of microsatellite data reveal a close affinity between the eastern and central populations (Table A4) and a clearly more differentiated western population (Hafner and Upham 25). Divergence following isolation may partly explain these genetic differences.

Our results indicate that since their divergence there has been effectively no gene flow among the eastern, central, and western populations of *M. megacephalus* (Table 2). Previous molecular evidence using mtDNA data suggests that lineage divergence within *M. megacephalus* occurred in the Pliocene, ~4 million years ago (Ma; Hafner and Upham 25; Hafner et al. 28), and fossil evidence from the late Blancan (1.9–2.9 Ma) supports that kangaroo mice diversified prior to the Pleistocene outside the Great Basin Desert. The significant difference between *R*_ST_ and *F*_ST_ values (Table A4) suggests that the populations have been isolated for a sufficiently long period of time such that mutation has played a relatively important role in genetic differentiation. The lack of significant differences among our historical *θ* values, and *t* parameters significantly larger than zero, suggest that each lineage may have diverged from a single ancestral population (Table 3). These results fail to reject the hypothesis that multiple lineages of *M. megacephalus* diverged from a common ancestral population and that some or all of these lineages invaded the Great Basin Desert in the early Pleistocene (coincident with the formation of appropriate sandy habitats; see Hafner and Upham 25 and references therein). Interestingly, this early Pleistocene colonization has been observed in other Great Basin taxa, such as pikas, brown creepers, and mountain chickadees (Grayson 22; Spellman et al. 63; Manthey et al. 46). It is important to note that our IMa estimates of *t* are significantly more recent than divergence times estimated in previous studies (Hafner et al. 28; Hafner and Upham 25). This discrepancy may be due to a complicated biogeographic history of the region making it difficult to track species history, sex-biased dispersal, and associated complications of using different genetic markers.

Although our demographic analyses postdate the Pliocene–Pleistocene transition (Fig. 4), we do observe a fairly constant population size over the past 200,000 years followed by possible recent population expansions. The recent expansion within the *M. megacephalus* central population is strongly supported by previous studies using mtDNA Bayesian Skyline Plots (BSP; Light et al. 42) and directional analyses of phylogeographic patterns (DAPP; Hafner and Upham 25). The recent expansions within the *M. megacephalus* eastern and western populations are not as strongly supported in previous analyses (Hafner and Upham 25; Light et al. 42), but this may be attributed to incomplete lineage sorting. Additionally, a constant population size may seem unlikely over a time period filled with climatic oscillations. Therefore, it seems more reasonable that the data do not contain enough demographic signals for these past events. Furthermore, it is important to note that due to excessively long computation times all EBSP analyses were performed using a simple model of evolution. Future studies comparing the results of more complex models of evolution (although previous studies note that skyline plots can be similar regardless of the model used; Allen et al. 1) and assessing the utility of EBSP analyses on microsatellite data should be performed.

Lack of evidence for current gene flow (Table 4), significant differences in microsatellite allele and genotype distributions, and previously documented reciprocal monophyly among *M. megacephalus* populations using mtDNA data (Hafner and Upham 25) support the view that each population, at the very least, should be managed as an evolutionarily significant unit. As noted by Hafner and Upham (25), the populations are very similar morphologically and are distributed in an allopatric manner (each population is separated by unsuitable habitat or geological barriers). Despite evidence for recent expansions, *N*_e_ estimates for all populations had lower bounds of confidence intervals and point estimates <500 (Table 3). As an *N*_e_ > 50 is needed to avoid inbreeding and an *N*_e_ > 500 to avoid extinction due to an inability to cope with environmental change (Franklin 20; Jamieson and Allendorf 38), our results suggest that these populations may be unable to adapt to environmental change and could be at risk for extirpation (Franklin 20). It is important to note that the exact *N*_e_ required for both long- and short-term sustainability has been disputed, and the minimum *N*_e_ may be higher than 50 (Nunney and Campbell 48; Lande 41), and the appropriate *N*_e_ may vary among populations (Flather et al. 18). Regardless, measures must be taken to conserve each genetically distinct lineage with appropriate management techniques for each population.

Similarly, the eastern and western *M. pallidus* populations are genetically distinct units that likely diverged ~4 Ma (Hafner et al. 28) with effectively no gene flow (far less than one migrant per generation) between them. Again, it is important to note that our IMa estimates of *t* are significantly more recent than divergence times estimated in previous studies (Hafner et al. 28), possibly due to a variety of reasons (see above). Similar to *M. megacephalus*, it is possible that one panmictic ancestral population (supported by our homogenous *θ* estimates, and positive *t* estimate) diverged outside of the Great Basin Desert and two independent lineages invaded the region at the beginning of the Pleistocene (supported by the significant difference between *R*_ST_ and *F*_ST_ values [Table A4]). The series of mountain chains that currently serve as a physiographic baffle between the eastern and western populations, likely prevented historic gene flow between these two lineages allowing for further divergence. Demographic analyses also suggest the *M. pallidus* western population has undergone a recent population contraction while the eastern population has remained constant, or has possibly undergone a population expansion, indicating that these two populations have historically been demographically independent from each other. Demographic results based on EBSP, however, should be interpreted cautiously (see above).

The lower bounds and point estimates of *N*_e_ of both the eastern and western populations of *M. pallidus* are well below 500 (Franklin 20); the western population even has an upper bound below 500 (Table 3). While these estimates may seem low, similar results have been found in other terrestrial vertebrates, some of whom are endangered (Nunney 47; Nunney and Campbell 48; Frankham 19; Phillipsen et al. 49; Hurtado et al. 37). Additionally, the small *N*_e_ of the western population is consistent with results from this study and a previous study using mtDNA, both indicating a recent population contraction (Light et al. 42). Thus, both the eastern and western populations may be in danger of extirpation and separate management practices for each population should be enforced (Traill et al. 64). To adequately measure the risk of extirpation, it will be important to further assess census sizes for both *M. megacephalus* and *M. pallidus*, which may be 2–10 times larger than these effective population size estimates (Nunney 47; Nunney and Campbell 48; Frankham 19).

### Broad implications

The amount of available habitat within the Great Basin Desert is decreasing as a result of a variety of anthropogenic alterations, and future climate change is predicted to reduce available habitat even further. Chaplin et al. (10) ranked the Great Basin as second in number of imperiled species among ecoregions of the United States. Habitat loss through agricultural practices, wildfires, and invasive plants has devastated the low-elevation areas where kangaroo mice from the eastern and western populations of *M. megacephalus* are distributed. Recent attempts to trap dark kangaroo mice from northern localities where mice were once abundant have been unsuccessful (J. C. Hafner, unpubl. data). Furthermore, repeated efforts to collect *M. pallidus* in once fruitful areas have either proven to be increasingly difficult or completely unsuccessful (Hafner et al. 28; J. C. Hafner, unpubl. data). As rare and highly specialized members of the Great Basin Desert, *Microdipodops* likely serve as indicator species of a healthy sandy desert ecosystem (Light et al. 42). Reduction in *Microdipodops* abundance may signal deterioration of the habitat, and further reduction in their abundance may prove detrimental to the survival of individual populations. The genus *Microdipodops* is a rare and highly specialized endemic of the Great Basin Desert, and this study provides further support that management and conservation efforts should be applied to each population in an effort to conserve these valuable taxa and the imperiled habitats of the Great Basin Desert.

## Data Accessibility

Data deposited in the Dryad repository: doi: 10.5061/dryad.r531f.

## Appendix 1

**Table:** Specimens and localities examined in this study. Localities listed by mtDNA clade identified in previous studies and then alphabetically by general locality^1^

Locality	*n*^2^	Museum vouchers^3^
*Microdipodops megacephalus*		
**Eastern clade:**		
Beryl: 0.7 mi N, 6.3 mi E Beryl, 5125 ft, Iron Co., Utah	8	MLZ 2145–2152
Callao: 7.7 mi S, 2.7 mi E Callao, 4500 ft, Juab Co., Utah	1	MSB 35599
Callao: 5.5 mi S, 7.8 mi E Callao, 4400 ft, Juab Co., Utah	1	MSB 35602
Geyser: 5.3 mi S, 1.6 mi E Geyser, 5900 ft, Lincoln Co., Nevada	2	MLZ 1974, 1975
Geyser: 5.2 mi S, 1.9 mi E Geyser, 5900 ft, Lincoln Co., Nevada	4	MLZ 1976–1979
Geyser: 5.1 mi S, 2.3 mi E Geyser, 5900 ft, Lincoln Co., Nevada	4	MLZ 1980–1983
Milford: 16.1 mi S, 19.6 mi E Garrison, 5400 ft, Millard Co., Utah	3	MLZ 2079–2081
Milford: 19.3 mi S, 18.4 mi E Garrison, 5100 ft, Millard Co., Utah	6	MLZ 2082–2087
Milford: 11.2 mi N, 39.6 mi W Milford, 5200 ft, Beaver Co., Utah	1	MLZ 2088
Minersville: 4.2 mi S, 15.8 mi W Minersville, 5050 ft, Beaver Co., Utah	8	MLZ 2071–2078
Minersville: Escalante Desert, 380 09.118'N, 1130 12.94'W, 1540 m, Beaver Co., Utah	2	BYU 30100, 30101
Osceola: 6.0 mi S, 4.2 mi W Osceola, 5800 ft, White Pine Co., Nevada	3	MLZ 1942–1944
Panaca: 24 mi W Panaca, 4600 ft, Lincoln Co., Nevada	4	MLZ 1752–1755
Pony Springs: 9.0 mi N, 10.8 mi W Pony Springs, 6020 ft, Lincoln Co., Nevada	2	MLZ 2059, 2060
**Central clade:**		
Austin: 6.2 mi S, 19.6 mi W Austin, 6150 ft, Lander Co., Nevada	4	MLZ 1748–1751
Belmont: 3.2 mi N, 4.2 mi E Belmont, 7000 ft, Nye Co., Nevada	4	MLZ 2027–2030
Benton: 5 mi N Benton, 5600 ft, Mono Co., California	6	MLZ 1740–1742 MLZ 1915–1917
Cherry Creek: 7.2 mi N, 8.8 mi E Cherry Creek, 5850 ft, White Pine Co., Nevada	1	MLZ 1965
Cobre: 0.9 mi S, 0.4 mi W Cobre, 5900 ft, Elko Co., Nevada	2	MLZ 2067, 2068
Contact: 10.9 mi S, 2.5 mi W Contact, 5700 ft, Elko Co., Nevada	2	MLZ 2069, 2070
Currant: 4.9 mi S, 28.2 mi W Currant, 6000 ft, Nye Co., Nevada	2	MLZ 2005, 2006
Danville: 6.1 mi S, 2.4 mi E Danville, 6800 ft, Nye Co., Nevada	3	MLZ 2021–2023
Duckwater: 8.4 mi N, 17.5 mi W Duckwater, 6350 ft, Nye Co., Nevada	3	MLZ 1997–1999
N Eureka: 22.8 mi N, 3.6 mi W Eureka, 5850 ft, Eureka Co., Nevada	4	MLZ 1956, 1957 MSB 35526, 35527
W Eureka: 6.2 mi N, 9.5 mi W Eureka, 6000 ft, Eureka Co., Nevada	2	MLZ 2031, 2032
Fletcher: 1/4 mile N Fletcher, 6100 ft, Mineral Co., Nevada	2	MLZ 1744, 1745
Goldfield: 12.0 mi N, 2.5 mi W Goldfield, 4860 ft, Esmeralda Co., Nevada	1	MLZ 1747
Gold Reed: 2.9 mi S, 3.1 mi E Gold Reed, 5350 ft, Nye Co., Nevada	1	MLZ 2053
Gold Reed: 2.9 mi S, 4.0 mi E Gold Reed, 5330 ft, Nye Co., Nevada	5	MLZ 2054–2058
N Hiko: 31 mi N, 1 mile W Hiko, 5100 ft, Lincoln Co., Nevada	1	MLZ 1960
W Hiko: 6 mi N, 31 mi W Hiko, 4800 ft, Lincoln Co., Nevada	2	MLZ 1815, 1816
Ruby Valley: 13.2 mi S, 0.6 mi E Ruby Valley, 6000 ft, Elko Co., Nevada	1	MLZ 2033
San Antonio: 3.7 mi N, 3.2 mi E San Antonio, 5600 ft, Nye Co., Nevada	2	MLZ 1761, 1762
Sunnyside: 1.3 mi S, 4.9 mi W Sunnyside, 5200 ft, Nye Co., Nevada	1	MLZ 1966
NE Tonopah: 13.8 mi N, 7.9 mi E Tonopah, 5800 ft, Nye Co., Nevada	4	MLZ 1961–1964
SE Tonopah: 9.8 mi S, 9.9 mi E Tonopah, 5200 ft, Nye Co., Nevada	1	MLZ 1831
Tybo: 1.0 mi N, 8.5 mi W Tybo, 6200 ft, Nye Co., Nevada	2	MLZ 1799, 1800
Warm Springs: 5.9 mi N, 10.2 mi E Warm Springs, 5200 ft, Nye Co., Nevada	1	MLZ 2024
Warm Springs: 6.4 mi N, 10.1 mi E Warm Springs, 5200 ft, Nye Co., Nevada	1	MLZ 2025
Warm Springs: 7.7 mi N, 9.5 mi E Warm Springs, 5200 ft, Nye Co., Nevada	1	MLZ 2026
NE Warm Springs: 19.2 mi N, 13.4 mi E Warm Springs, 6000 ft, Nye Co., Nevada	5	MLZ 1905 MLZ 1948–1951
SE Warm Springs: 12.7 mi S, 0.4 mi E Warm Springs, 6000 ft, Nye Co., Nevada	5	MLZ 1968–1972
**Western clade:**		
Chilcoot: 1.7 mi N Chilcoot, 5100 ft, Plumas Co., California	1	MLZ 1756
Chilcoot: 1.5 mi N Chilcoot, 5100 ft, Plumas Co., California	1	MVZ 158930
Denio: 0.6 mi S Denio, 4200 ft, Humboldt Co., Nevada	2	MSB 35530, 35531
Fields: 2.4 mi N, 3.4 mi E Fields, 4050 ft, Harney Co., Oregon	9	MLZ 2007–2015
Gerlach: 28.5 mi N, 27.8 mi W Gerlach, 4700 ft, Washoe Co., Nevada	5	MLZ 2089–2093
Gerlach: 28.2 mi N, 27.6 mi W Gerlach, 4700 ft, Washoe Co., Nevada	5	MLZ 2094–2098
Gerlach: 24.5 mi N, 25.0 mi W Gerlach, 4800 ft, Washoe Co., Nevada	1	MLZ 2099
Gerlach: 24.0 mi N, 24.8 mi W Gerlach, 4800 ft, Washoe Co., Nevada	5	MLZ 2100–2104
Gerlach: 22.4 mi N, 23.6 mi W Gerlach, 4800 ft, Washoe Co., Nevada	5	MLZ 2105–2109
Jungo: 13.8 mi N, 11.2 mi E Jungo, 4200 ft, Humboldt Co., Nevada	5	MLZ 2124–2128
Ravendale: 4.4 mi N, 13.6 mi E Ravendale, 5650 ft, Lassen Co., California	2	MLZ 2110, 2112
Ravendale: 4.7 mi N, 10.8 mi E Ravendale, 5350 ft, Lassen Co., California	2	MLZ 2113–2114
Sparks: 6 mi N, 4 mi E Sparks, 4600 ft, Washoe Co., Nevada	3	MLZ 1757–1759
Valley Falls: 36 mi N, 14 mi E Valley Falls, 4300 ft, Lake Co., Oregon	10	MLZ 1987–1996
Vernon: 0.5 mi S, 11.5 mi W Vernon, 4450 ft, Pershing Co., Nevada	1	MLZ 1760
Vya: 3.2 mi N, 11.5 mi E Vya, 5600 ft, Washoe Co., Nevada	3	MLZ 1984–1986
N Winnemucca: 7 mi N Winnemucca, 4600 ft, Humboldt Co., Nevada	1	MSB 35533
SW Winnemucca: 5.5 mi S, 9.2 mi W Winnemucca, 4300 ft, Humboldt Co., Nevada	1	MSB 35535
**Idaho clade:**		
Riddle: Starr Valley, NW ¼ Section 19, T16S, R5W, B.M., Owyhee Co., Idaho	1	IMNH 259
Riddle: 1/2 mi N Nevada, 2 1/2 mi E Oregon, Owyhee Co., Idaho	1	IMNH 693
Riddle: 11 mi S, 44.2 mi W Riddle, 5000 ft., Owyhee Co., Idaho	2	MLZ 2163–2164
*Microdipodops pallidus*		
**Eastern clade:**		
Alamo: 4.5 mi S, 32.5 mi W Alamo, 4600 ft, Lincoln Co., Nevada	1	MSB 35536
Currant: 4.9 mi S, 28.2 mi W Currant, 6000 ft, Nye Co., Nevada	5	MLZ 2000–2004
Goldfield: 12.0 mi N, 2.5 mi W Goldfield, 4860 ft, Esmeralda Co., Nevada	2	MLZ 1743, 1746
SE Goldfield: 4.6 mi S, 19.8 mi E Goldfield, 4950 ft, Nye Co., Nevada	2	MLZ 2051, 2052
Gold Reed: 3.0 mi S, 4.3 mi E Gold Reed, 5330 ft, Nye, Co., Nevada	2	MLZ 1958, 1959
W Hiko: 6 mi N, 31 mi W Hiko, 4800 ft, Lincoln Co., Nevada	4	MLZ 1811–1814
Lockes: 9.6 mi S, 3.8 mi W Lockes, 4800 ft, Nye Co., Nevada	4	MLZ 2017–2020
New Reveille: 0.9 mi N, 10.3 mi E New Reveille, 4900 ft, Nye Co., Nevada	2	MLZ 1940–1941
San Antonio: 0.5 mi S San Antonio, 5400 ft, Nye Co., Nevada	1	MLZ 1798
E Tonopah: 0.5 mi N, 32.0 mi E Tonopah, 5600 ft, Nye Co., Nevada	4	MLZ 1801–1804
SE Tonopah: 11.0 mi S, 10.0 mi E Tonopah, 5200 ft, Nye Co., Nevada	5	MLZ 1821–1825
SE Tonopah: 10.6 mi S, 10.0 mi E Tonopah, 5200 ft, Nye, Co., Nevada	5	MLZ 1826–1830
NE Warm Springs: 19.2 mi N, 13.4 mi E Warm Springs, 6000 ft, Nye Co., Nevada	5	MLZ 1906, 1952–1955
**Western clade:**		
Coaldale: 1.8 mi S, 5.3 mi E Coaldale, 4797 ft, Esmeralda Co., Nevada	1	MLZ 1817
Deep Springs: 7.2 mi S, 4.0 mi W Deep Springs, 4920 ft, Inyo Co., California	2	MLZ 1767, 1768
Deep Springs: 4.6 mi S, 3.9 mi W Deep Springs, 5000 ft, Inyo Co., California	2	MLZ 1769, 1770
Deep Springs: 2.4 mi S, 2.3 mi W Deep Springs, 5050 ft, Inyo Co., California	6	MLZ 1771–1776
Dyer: 7.0 mi N, 0.5 mi W Dyer, 4900 ft, Esmeralda Co., Nevada	5	MLZ 1785–1789
Fallon: 4.3 mi N Fallon, 3900 ft, Churchill Co., Nevada	3	MLZ 1947, 2115–2116
Lovelock: 2.5 mi N, 22.5 mi W Lovelock, 3950 ft, Pershing Co., Nevada	3	MLZ 1967, 2117–2118
Luning: 9.8 mi N, 10.8 mi E Luning, 5350 ft, Mineral Co., Nevada	5	MLZ 1805–1809
Luning: 12.7 mi N, 9.2 mi E Luning, 5050 ft, Mineral Co., Nevada	1	MLZ 1810
Marietta: 0.4 mi S, 0.5 mi E Marietta, 4950 ft, Mineral Co., Nevada	3	MLZ 1777–1779
Mina: 8.9 mi S, 1.2 mi E Mina, 4400 ft, Mineral Co., Nevada	10	MLZ 1780–1784 MLZ 2119–2123
Nixon: 6.4 mi N, 1.0 mi W Nixon, 4200 ft, Washoe Co., Nevada	1	MLZ 1794
Oasis: 0.2 mi S, 1.5 mi E Oasis, 5050 ft, Mono Co., California	2	MLZ 1790, 1791
Oasis: 1.0 mi S, 4.0 mi E Oasis, 5100 ft, Mono Co., California	2	MLZ 1792, 1793
San Antonio: 0.5 mi S San Antonio, 5400 ft, Nye Co., Nevada	2	MLZ 1796–1797
Schurz: 7.3 mi N, 2.6 mi W Schurz, 4287 ft, Mineral Co., Nevada	3	MLZ 1818–1820
Silver Peak: 5.1 S, 1.1 mi E Silver Peak, 4300 ft, Esmeralda Co., Nevada	2	MLZ 1945, 1946
NW Tonopah: 9.2 mi N, 8.1 mi W Tonopah, 4850 ft, Nye Co., Nevada	1	MLZ 1973
Wadsworth: 1.0 mi N, 1.0 mi W Wadsworth, 4200 ft, Washoe Co., Nevada	1	MLZ 1795
Yerington: 11.7 mi S, 3.5 mi E Yerington, 4690 ft, Lyon Co., Nevada	3	MLZ 1832–1834
Yerington: 11.1 mi S, 2.8 mi E Yerington, 4640 ft, Lyon Co., Nevada	5	MLZ 1835–1839

1 Localities are plotted on distribution maps in Hafner et al. (28), Hafner and Upham (25), and Light et al. (42).

2 Number of samples.

3 Museum abbreviations are as follows: Moore Laboratory of Zoology (MLZ, Occidental College), Museum of Southwestern Biology (MSB, University of New Mexico), Monte L. Bean Life Science Museum (BYU, Brigham Young University), San Diego Natural History Museum (SDNHM), Idaho Museum of Natural History (IMNH, Idaho State University), and the Museum of Vertebrate Zoology (MVZ, University of California, Berkeley).

## Appendix 2

**Table:** Summary statistics of 11 microsatellite loci found within *Microdipodops megacephalus* from the eastern, central, western, and Idaho geographic units

	*Mime2*	*Mime3*	*Mime11*	*Mime12*	*Mime21*	*Mime24*	*Mime29*	*Mime32*	*Mime33*	*Mime35*	*Mime36*
Eastern unit											
N	49	49	49	49	49	49	48	48	49	49	47
H_O_	0.91837	0.73469	0.67347	0.7551	0.85714	0.77551	0.72917	0.72917	0.77551	0.87755	0.76596
H_E_	0.84683	0.83947	0.80391	0.8077	0.88239	0.73175	0.76425	0.77697	0.80454	0.80623	0.80599
HW	0.62137	0.01491	0.0108	0.0097	0.06952	0.0209	0.2349	0.33228	0.37365	0.10825	0.18306
A	9	8	9	12	14	6	9	8	9	7	9
A_R_	4.17	4.097	3.819	3.971	4.539	3.244	3.553	3.598	3.886	3.789	0.5625
Central unit											
N	68	69	69	69	69	68	66	68	68	69	69
H_O_	0.83824	0.71014	0.72464	0.76812	0.82609	0.86765	0.74242	0.82353	0.80882	0.75362	0.81159
H_E_	0.85261	0.83233	0.79816	0.81847	0.85507	0.79869	0.79517	0.8244	0.84564	0.78536	0.88681
HW	0.45032	0.02758	0.0832	0.56024	0.56024	0.47868	0.26346	0.59097	0.25529	0.66839	0.44048
A	11	10	8	10	13	7	9	8	10	8	14
A_R_	4.248	4.033	3.751	3.954	4.26	3.737	3.735	3.983	4.187	3.684	4.586
Western unit											
N	59	62	62	62	61	61	58	59	61	62	61
H_O_	0.79661	0.79032	0.77419	0.74194	0.77049	0.85246	0.84483	0.84483	–	0.62903	0.63934
H_E_	0.80878	0.80698	0.76882	0.81655	0.88227	0.87197	0.88516	0.88516	–	0.72712	0.79217
HW	0.16764	0.44102	0.04119	0.15546	0.00669	0.19183	0.06831	0.06831	–	0.1071	0.07473
A	9	9	8	8	14	9	17	7	1	6	8
A_R_	3.869	3.864	3.533	3.958	4.533	4.396	4.598	3.288	1	3.261	3.718
Idaho unit											
N	4	4	4	4	4	4	4	4	4	3	3
H_O_	1	1	0.5	0.5	0.75	0.25	1	1	–	1	0.667
H_E_	0.67857	0.71429	0.67857	0.42857	0.75	0.46429	0.75	0.89286	–	0.6	0.8
HW	0.31266	0.5433	1	1	1	0.14273	1	1	–	0.39954	0.60073
A	3	3	3	2	4	3	3	5	1	2	4
A_R_	2.75	2.929	2.75	1.964	3.464	2.5	2.964	4.393	1	2	4

N, number of individuals; H_O_, observed heterozygosity; H_E_, expected heterozygosity; HW, probability of conformance to Hardy–Weinberg equilibrium; A, number of alleles; A_R_, allelic richness.

## Appendix 3

**Table:** Summary statistics of 10 microsatellite loci found within *Microdipodops pallidus* from the eastern and western geographic units

	*Mime2*	*Mime4*	*Mime5*	*Mime11*	*Mime12*	*Mime24*	*Mime29*	*Mime32*	*Mime33*	*Mime35*
Eastern unit										
N	42	41	42	42	42	42	41	42	42	42
H_O_	0.69048	0.73171	0.7381	0.7619	0.80952	0.8333	0.85366	0.54762	0.38095	0.7381
H_E_	0.82387	0.75008	0.73896	0.80034	0.79231	0.79346	0.90003	0.71572	0.31211	0.77653
HW	0.50682	0.99956	0.64144	0.17348	0.47011	0.49504	0.07527	0.12538	0.31231	0.80186
A	7	8	6	9	9	7	13	6	2	7
A_R_	6.976	8	5.976	8.952	8.952	6.976	13	5.976	2	7
Western unit										
N	62	63	57	56	63	62	63	63	63	63
H_O_	0.64516	0.77778	0.89474	0.78571	0.69841	0.838971	0.77778	0.65079	0.04762	0.77778
H_E_	0.75138	0.78756	0.90545	0.88095	0.80698	0.89156	0.76825	0.77168	0.07759	0.75949
HW	0.01113	0.87267	0.03936	0.00136	0.052	0.13845	0.2815	0	0.03251	0.19073
A	9	7	17	12	8	15	11	7	3	7
A_R_	8.496	6.302	16.522	11.194	7.946	14.485	9.809	6.301	2.839	6.945

N, number of individuals; H_O_, observed heterozygosity; H_E_, expected heterozygosity; HW, probability of conformance to Hardy–Weinberg equilibrium; A, number of alleles; A_R_, allelic richness.

## Appendix 4

**Table:** Table of pairwise genetic distances and 95% confidence intervals among geographic units, including results of the allele size permutation test, calculated in SPAGeDi 1.4^1^

Population pair permutation test	*F*_ST_	*R*_ST_	Allele size (*R*_ST_ = *F*_ST_)
*M. megacephalus*			
Eastern vs. Central units	0.03 (0.028)	0.14 (0.12)	*P *<* *0.001
Eastern vs. Western units	**0.11 (0.12)**	0.70 (0.36)	*P *<* *0.001
Eastern vs. Idaho units	0.20 (0.08)	0.69 (0.27)	*P *<* *0.001
Central vs. Western units	**0.11 (0.11)**	0.64 (0.30)	*P *<* *0.001
Central vs. Idaho units	0.17 (0.08)	0.60 (0.22)	*P *=* *0.076^2^
Western vs. Idaho units	0.10 (0.06)	0.22 (0.21)	*P *=* *0.063^2^
*M. pallidus*			
Eastern vs. Western units	0.08 (0.04)	0.91 (0.33)	*P *<* *0.001

1 Genetic distances are slightly, but not significantly, different from Arlequin results (see text).

2 Although 95% confidence intervals suggest significance, small sample sizes for Idaho likely resulted in nonsignificant results for the allele size permutation tests.

Values in bold are not significantly different from zero.
